# Motion shapes for sound shaping

**DOI:** 10.3389/fpsyg.2024.1449021

**Published:** 2024-08-16

**Authors:** Rolf Inge Godøy

**Affiliations:** RITMO Centre for Interdisciplinary Studies in Rhythm, Time and Motion, Department of Musicology, University of Oslo, Oslo, Norway

**Keywords:** sound, motor control, multimodality, intermittency, motion

## Abstract

The focus of this perspective paper is on relationships between sound-producing body motion and corresponding perceived sound features, guided by the idea of shapes as the common denominator of these two domains. The term *shape* is used to denote graphical-pictorial renderings of phenomena that we perceive or imagine, and may have physical manifestations as tracings on paper or on screen, or as gesticulations, or just as imagined tracings in our minds. Shapes give us intermittent snapshots of unfolding motion and sound fragments, and the point of shapes is to make ephemeral sound and motion features tractable as more permanent objects. Shapes of perceived sound include dynamic, spectral, textural, pitch-related, harmonic, etc. features as shapes, whereas shapes of sound-producing motion include both motion trajectories and postures of sound-producing effectors, i.e., of fingers, hands, arms, etc., or mouth, lips, and tongue.

## 1 Introduction

As suggested by the topic of networked music perception and production, music seems to be distributed across several human faculties, requiring close cooperation and synchrony of the involved faculties. Yet in spite of this distributed basis, it seems that music is also robust and capable of evoking spontaneous body motion responses independent of perceivers’ expertise, e.g., as evident in cases of so-called *entrainment*, with perceivers making spontaneous body motion in sync with some salient feature of the music, such as in dancing, walking, nodding head, or gesticulating (Clayton et al., 2013).

Such perceivers’ motion may resemble the sound-producing motion of the performers, be that as seen at a concert or only as imagined when listening to recordings. Perceivers seem to have extensive knowledge of such sound-motion associations, as attested to by cases of so-called *air instrument performance* (Godøy et al., 2006). I have previously used the expression *motormimetic cognition* for this sensation of sound-producing motion (Godøy, 2001, 2003), inspired by the so-called *motor theory of perception*, a theory which links auditory sensations in speech with motion sensations of the vocal apparatus (Galantucci et al., 2006).

In this paper, the focus will be on the details of such perceived and/or imagined sound-producing motion, with the motivation that these are salient sensations reflecting both the energy of the music, and variably so, the more small-scale effector motion and postures that go into sound-production. The motivation here is that systematic knowledge of sound-producing motion can enhance our knowledge of musical features in general, and also have practical applications, given the close connection between such motion and output sound features.

Furthermore, both the sound-producing body motion and the resultant sound, can be represented by shape images, making the otherwise ephemeral motion and sound features available for closer scrutiny as more permanent shape images, thanks to now readily available methods and technologies. What we may collectively call *shape cognition* is then not only a tool for studying motion and sound separately, but also a tool for studying the links between these two domains, hence the idea here of studying *motion shapes for sound shaping*.

We shall now first consider some relevant issues of multimodality, motion typology, and associated motion constraints, before going on to intermittency, i.e., discontinuity in motor control and sound perception, as well as what we call *sound-motion objects*, followed by a concluding discussion of challenges in motor control research on motion and sound shapes.

## 2 Intrinsic multimodality

**Figure 1:**
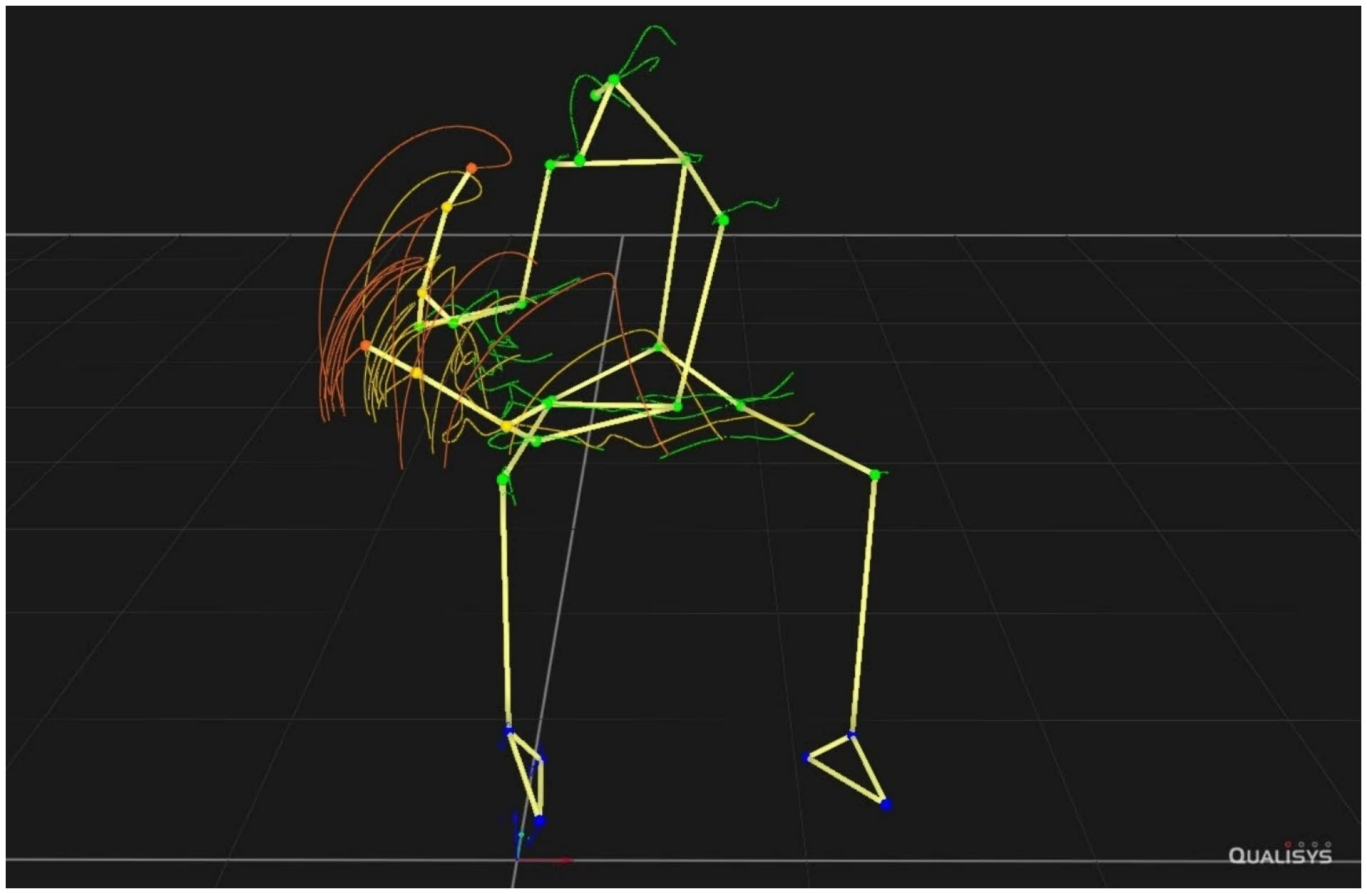
Motion capture tracing of a fast drum fill performed across a drumset, see main text for details.

Consider an expert drummer playing a fast drum fill across the drumset from left to right, necessitating both a rush of fast mallets-hands-arms striking motion, and a turn of the torso from left to right to reach the target instruments, as may be seen from the motion capture traces in Figure 1 (see Godøy et al., 2017 for details). Obviously well practiced, this entire fill may be perceived as a coherent multidimensional sound-producing motion shape as well as a rush of drum sounds, and raises the question of what this is: Is it a matter of motion perception or sound perception, or a combination of both? In the latter case, this would be an example of intrinsic multimodality, of motion and sound fused into what we call a *sound-motion object*.

Needless to say, memory for sound-producing motion is the *sine qua non* for performers, and mental practice involving imagined sound-producing motion is regarded as an efficient method by many musicians (Bernardi et al., 2013; Lotze, 2013). Also, in cases of improvised music, we have accounts of performers letting memory of sound-producing motion guide the sound output (Sudnow, 1978). In short, we see sound-producing motion shapes and postures everywhere, testifying to the intrinsic multimodality of sound and motion in music, motion in turn consisting of several modalities, prominently so vision, proprioception, and haptics. It would not be too farfetched to state that very many, if not most musical features, are indeed multimodal (Godøy et al., 2016).

The basic tenet in this paper is to consider music as a *concrete* phenomenon, concrete as opposed to *abstract,* as advocated by Schaeffer (2017) and Godøy (2021a), concrete here signifying the distributed sound substrate of music, i.e., music as based on *sound objects,* i.e., time-limited fragments of sound, be that actual audio signals or mental images of such audio signals. A crucial element here is that sound objects are coherent entities, conforming to Schaeffer’s criteria of so-called *suitable objects*, meaning within the approximate duration range of 0.5 to 5 s, hence, not too short nor too long, as well as not too varied, nor too static, in brief, being what Schaeffer and co-workers considered *balanced* (Schaeffer, 2017). And importantly, sound objects can be represented as shapes, notably so as shapes of different concurrent features (dynamic, spectral, pitch-related) and at different timescales, from the overall dynamic shape of the entire sound object down to minute fluctuations within the sound object, and that all these shapes are motion-related (Godøy et al., 2006).

The significant advantage of this approach is that perceptually salient features, not well represented by traditional Western notation-based symbols, can be represented by shape images, be that as graphical images and/or as motion-related tracings. Crucially, shapes are inherently holistic in the sense that they are images of the entire entity in question, as is the main message of the so-called *morphodynamic theory* of perceptually salient features (Thom, 1983; Petitot, 1990).

## 3 Motion typology

Music-related body motion may be classified as either *sound-accompanying* or as *sound-producing* (Godøy and Leman, 2010). Sound-accompanying motion typically denotes the listeners’ body motion in sync with the beat of the music and/or reflecting the overall energy and motion mode of the music, whereas the sound-producing motion denotes the perceived (or imagined) motion of the sound-producing effectors, e.g., fingers, hands, arms, etc. on instruments and in sync with the details in the output sound. The main feature of sound-producing motion (notably in non-electronic music), is that of transferring energy from the body to a musical instrument to produce audible sound, by striking, plucking, bowing, blowing, stroking, kicking, etc. as well as for modifying the output sound, such as by changing the pitch by moving the left hand on string instruments, changing timbre by moving the bowing point, or by opening and closing of mutes, etc.

There are some crucial distinctions in the motion types, based on the main categories of *impulsive*, *sustained*, and *iterative* suggested by Pierre Schaeffer for classifying sound objects within the so-called *musique concrète* (Schaeffer, 2017), but which turn out to also encompass distinct, and mutually exclusive, modes of motion, e.g., it is not possible to make impulsive motion that is sustained, or sustained motion that is iterative. However, it is possible to have transitions between categories by changing the duration or rate of motion, e.g., lengthening the duration of an impulsive motion turns it into a sustained motion, increasing the rate of an impulsive motion turns it into an iterative motion, etc., hence, to make category changes that demonstrate so-called *phase transition* (Haken et al., 1985).

There are several other motion features linked with salient sound features, such as overall energy level of the motion, what may be referred to as *quantity of motion*, due to combined levels of motion amplitude and rate, as well as the motion derivatives such as *acceleration* and *jerk*, indicating perceptually salient features of roughness vs. smoothness of motion (Gonzalez-Sanchez et al., 2019).

Crucially, various pitch-related motion, e.g., the fingers/hands motion along the neck of a string instrument or along the keyboard of a piano, as well as timbre-related motion, e.g., opening and closing of a trumpet mute, the *sul pont* to *sul tasto* motion of the bowing on a violin, or the changing vowel shapes of the vocal apparatus in singing, all reflect salient and, we may assume, for very many perceivers, familiar sound-modifying kinds of motion.

In a motor control perspective, these time-dependent sound changes can then be mapped to motion shapes, shapes consisting of so-called *goal postures* (Rosenbaum, 2010), and these postures occurring at salient moments, at what we may call *goal points* in time.

## 4 Motion constraints

In line with the concrete approach to music cognition, it is useful to consider some constraints at work in making musical sound. There are the constraints of musical instruments, of the vocal apparatus, and of body motion, that limit what sound output is possible. But constraints can also engender workaround solutions that in turn may become integral to sound producing motion and the output sound. We shall now just briefly mention some main constraints of sound-producing motion and refer to other publications for instrumental-acoustic constraints features (e.g., Rossing, 2002). The main motion constraints are concerned with biomechanics, motor control, and need for optimalization. Crucially, some of these constraints will lead to intermittency, i.e., the discontinuity in effort and control, in turn related to various emergent features of sound-motion objects.

The first and most important constraint is that all motion takes time, i.e., that there is no instantaneous displacement of sound-producing effectors. From this rather obvious point, there are the consequences of workarounds by extensive use of preparatory motion, i.e., that prior to the onset of sound events, the effectors need to move to a position that is optimal for an upcoming event.

Known as *coarticulation*, this is a phenomenon found in several domains of human motion (Rosenbaum, 2009) and includes anticipatory motion in sound producing motion (Godøy, 2014). In addition to the effects of such anticipatory coarticulation, there is also the influence of the just past effector motion, a spillover effect of coarticulation, meaning that there is contextual smearing both forwards and backwards in time at work because of coarticulation. Additionally, coarticulation may by such anticipatory and spillover motion actually reduce the amount of needed motion (Sosnik et al., 2004), resulting in more smooth motion.

A related constraint here is that there are limits to speed, to effort, and endurance, so that musicians need to grasp moments of relaxation whenever possible to conserve energy and avoid strain injury. Energy conservation also means exploiting rebounds, and is related to intermittency, in that muscle contraction periods are minimized so as to enable interleaved periods of relaxation.

Also, we may observe constraint-based changes in motion modes, for instance in the case of acceleration of cello bowing motion where slow bowing allows for long bowing motion, frog to tip, but with increasing tempo, the bowing motion becoming shorter and shorter until becoming like a small amplitude tremolo motion at the fastest rate, and notably so, with corresponding timbral changes from predominantly harmonic to increasingly more noisy because more frequent bow direction changes produce more transient noise and spectral flux (Godøy, 2021b).

## 5 Intermittency in perception and production

Intermittency, defined as something occurring discontinuously, may be seen as a constraint-based feature for both perception and production in music. For perception, there is the issue, discussed by Husserl and contemporaries, of the need to step out of continuous streams of sensations in order to make sense of these sensations by accumulation into intermittent overview images. With the expression *now-points,* Husserl suggested that we perceive the streams of continuous sensations on a discontinuous, moment-by-moment basis, and that these now-points contain images of the recent past, of what’s going on at the moment, and importantly, of expectations of the future, making up Husserl’s famous tripartite model of *retentions, primary impressions,* and *protentions* (Husserl, 1991; Godøy, 2010).

More specifically for auditory perception, the so-called *cut bell* experience of Schaeffer and co-workers, i.e., of cutting off the attack phase of a bell sound making it become more like a flute sound, demonstrated that the sense of “belleness” required perceiving a longer stretch of sound (Schaeffer, 2017). Considered to be valid for sound objects in general, it was suggested that the mentioned 0.5 to 5 s duration range was optimal for most perceptual features, hence, required an intermittent perception of the entire sound object in question. Interestingly, we have also seen suggestions of a similar optimal duration around 3 s in other domains of perception, cognition, and also human motion (Pöppel, 1997).

In motion, recognizing the sluggishness of motor control and the so-called *psychological refractory period*, has engendered anticipatory cognition as a workaround solution with preplanning of motion to facilitate rapid motion chunks by avoiding time-consuming planning (Klapp and Jagacinski, 2011). Known in the intermittency literature by various terms such as *open loop*, *feedforward*, or *serial ballistic* control (Loram et al., 2014), the common feature is intermittency of control, similar to the point-by-point procedure for perception. A related instance of such point-by-point control, is that of a posture-by-posture control scheme, with the continuous motion between the postures subordinate to the postures (Rosenbaum, 2010, 2017). This posture-by-posture control scheme has the advantages of simplifying control, by way of a hierarchical and anticipatory control scheme, which in turn may lead to coarticulation and associated features of coherence and smoothness, hallmarks of skilled motion.

The idea of intermittency in motor control dates back to the seminal work of Kenneth Craik (1947) and Margaret Vince (1948), and has been elaborated in more recent publications (e.g., Ronco, 1999; Karniel, 2013; Loram et al., 2014), but needless to say, there are a number of not well understood elements here, in particular as to the contents of the intermittent control commands in human motion. But what seems already well-founded, is the principle of anticipatory cognition enabling fast and accurate execution of motion chunks. The mentioned shape images of motion could be the content of such anticipatory control, a “receding horizon” (Ronco, 1999) within a sound-motion object. Another interesting model is that of muscle synergies, i.e., a network-like scheme of low-level muscle activation and relaxation that facilitates high-level volitional motor control (d'Avella and Lacquaniti, 2013).

Although motor control is a rather diverse domain, there seem to be a consensus that some degree of preplanning is involved in skilled motion, but with quite differing opinions on how the triggering and control during motion actually works. One suggestion here is that of a so-called *initial impulse*, i.e., a spike of activation engendering rapid motion, sometimes referred to as *ballistic motion*, with more feedback control occurring after this initial impulse (Elliott et al., 2001). The idea of an initial triggering impulse is interesting in our context, as part of a generic impulse-response type model of intermittent energy input and intermittent control, together resulting in piecewise continuous sound-motion output (Godøy, 2013, 2022).

## 6 Sound-motion objects

Schaeffer’s music theory is based on subjective perception of sound objects as coherent entities with overall dynamic, pitch-related, and timbral features, and internal textural features, down to small-scale fluctuations (Schaeffer, 1998, 2017), all of which may be conceptualized as shapes. Although sound objects may be derived from environmental, electronic, and instrumental sources, their overall energy envelopes are closely linked with the sound-producing motion categories listed earlier, so it makes sense to expand the notion of sound objects to include motion, hence, the expression *sound-motion objects*.

The coupling of sound object with sensations of sound-producing motion, means that the various concrete constraints from our environment may serve to give such objects a stronger coherence, specifically by schemas of energy input and dissipation. Consider, e.g., the case of a tamtam struck with a soft mallet resulting in an initial impact sound followed by a lengthy energy dissipation phase, where the envelope of the white noise cloud is a shape from an impact event and its subsequent energy dissipation. Similar impulse-dissipation schemas, which may be generated by physical model synthesis, offer ecological plausible coherence that more abstract digital synthesis models do not.

Similar linking of motion envelopes with resultant output sound envelopes, are then at the core of this idea of motion shapes for sound shaping, and may be applied to most other cases of sound objects, e.g., motives, textural fragments, melodic contours, ornaments, etc., sound objects with the common feature of high levels of coherence and clear energy dissipation envelopes, as well as a high levels of preplanning exhibiting gestalt-like coherence (Klapp and Jagacinski, 2011).

## 7 Discussion: challenges in motor control research on motion and sound shapes

Hopefully, Western music-related research will in the coming years be more directed toward motor control, given current advances in methods and technologies for capturing, processing, and representing ephemeral motion and sound data, and all the more so, when such data may hold much salient perceptual information. The crux of the matter now is having conceptual tools for handling non-symbolic, emergent ephemeral information on motion and sound, something I believe is offered by shape cognition, *cf.* the mentioned morphodynamical theory combined with now readily available shape data from sound recording and motion capture (Godøy, 2021b).

Another challenge is understanding the constraints of motor control, in particular those related to intermittent anticipatory images of sound-motion objects. This means knowing more about how intermittent cumulative perceptual sensations, as well as intermittent anticipatory motor control images, actually work, including the relationships between continuous motion and stationary postures (Rosenbaum, 2017). Concerning intermittency and motor control in general, the least studied element seems to be that of triggering, i.e., of how preplanned motion chunks, all ready to go, are actually kicked into running their course.

Exploring further these elements of motion shapes related to emergent sound shapes, and in particular the topics of sound-motion objects and intermittent control, could very well be done in more non-traditional contexts such as various art forms, e.g., in dance, composition, improvisation, and sound design, allowing for a focus on non-symbolic emergent coherent shapes of motion and sound.

## Data Availability

The original contributions presented in the study are included in the article/supplementary material, further inquiries can be directed to the corresponding author.
